# Clinico-Epidemiological Profile and Treatment Pattern of Vitiligo in Selected Dermatological Clinics of Mekelle City, Northern Ethiopia

**DOI:** 10.1155/2020/3625753

**Published:** 2020-05-30

**Authors:** Afewerki Gebremeskel Tsadik, Goitom Fitsum Legesse, Desilu Mahari Desta, Brhane Teklebrhan Assefa, Hailekiros Gebretsadik Kidanemariam, Meles Tekie Gidey

**Affiliations:** ^1^Clinical Pharmacy Unit, School of Pharmacy, College of Health Sciences, Mekelle University, Mekelle, Tigray, Ethiopia; ^2^Student in School of Pharmacy, College of Health Sciences, Mekelle University, Mekelle, Tigray, Ethiopia; ^3^Department of Pharmacology, School of Pharmacy, College of Health Sciences, Mekelle University, Mekelle, Tigray, Ethiopia; ^4^Department of Pharmaceutical Analysis and Quality Assurance, School of Pharmacy, College of Health Sciences, Mekelle University, Mekelle, Tigray, Ethiopia; ^5^Pharmacoepidemiology and Social Pharmacy Course and Research Unit, School of Pharmacy, College of Health Sciences, Mekelle University, Mekelle, Tigray, Ethiopia

## Abstract

**Background:**

Vitiligo is not a well-studied disease in Ethiopia. Therefore, this study assessed its clinico-epidemiological profile and treatment patterns.

**Methods:**

An institutional-based cross-sectional study was conducted in conveniently selected dermatologic clinics of Mekelle city, Ethiopia. A two-phased study was conducted, in which the first was to determine prevalence of vitiligo while the second phase was to describe the clinico-epidemiological profile and treatment pattern of vitiligo. Four-hundred three randomly selected dermatological patients were included in the first phase study. The second phase study included vitiligo cases from the first phase study and additional vitiligo cases found in a two months period prospective study.

**Results:**

Of the 403 randomly selected dermatological patients who presented in the year 2017 to 2019, the prevalence of vitiligo was 13.15%. Of the 79 cases with vitiligo, nearly two-thirds (50, 63.3%) were males with five years as the median age at onset of the disease. Positive family history of vitiligo was recorded in about one-third (25, 31.6%) of the cases. Limbs (48, 44.5%) followed by the head and neck (26, 24%) were the most commonly affected parts of the body at the onset of the disease. The most prevalent clinical form of vitiligo was vulgaris (39.2%) followed by the focal type (26.6%). Emotional upset (24, 33.8%) and physical traumas (23, 32.4%) were the frequently reported triggering factors of vitiligo. Three-fourths (75.5%) of the cases had prescriptions of topical corticosteroids, and 24.5% of them had prescriptions of sun screen lotion.

**Conclusion:**

The prevalence of vitiligo was found to be high. The clinico-epidemiological profile of vitiligo in Ethiopia was similar with that found globally. However, treatment options of vitiligo were very limited in Ethiopia.

## 1. Introduction

Vitiligo is a common acquired, probably heritable, progressive depigmenting skin disorder characterized by destruction of melanocytes within the epidermis, the mucous membranes, the eyes, and occasionally in some hair bulbs [1]. The prevalence of vitiligo was 0.5–2% globally. Most commonly, the disease begins during childhood or young adulthood with onset of 10 to 30 years but can occur at any age [2, 3]. Vitiligo patches can appear anywhere on the skin, but common sites are usually around the orifices, the genitals, or sun-exposed areas such as the face and hands. In addition to white patches on the skin, people with vitiligo may have poliosis of the scalp hair, eyelashes, eyebrows, and beard [4]. The disease is classified as generalized or localized; according to its extent and distribution. A generalized type of Vitiligo clinically present as symmetrical depigmented patch. A less common type is the segmental form in which asymmetrical, focal, or dermatomal depigmentation patch or macules develop [5]. Vitiligo is a multifactorial polygenic disorder with complex pathogenesis; hence, several theories have been proposed to explain the loss of epidermal melanocytes like; genetic, immunological, biochemical (including oxidative stress), and neurogenic factors may interact to contribute to its development [6]. Additionally, certain factors have been identified like trauma to the skin, hormonal changes, and stress may be necessary for the disease to become apparent [7].

Vitiligo not only cosmetically disfigures the patient but also leads to psychological consequences. These effects range from mild embarrassment to a severe loss of self-confidence and social anxiety, especially for those who have lesions on exposed skin [8, 9]. Moreover, individuals with vitiligo suffer social discrimination and stigmatization, which result in significant changes in their lifestyles: from the choice of clothing, use of sunscreen, and cosmetic camouflage of the lesions to the avoidance of social events or outdoor activities [10–12].

The current treatments for Vitiligo are difficult, expensive, and often disappointing. The degree of repigmentation that defines success has been arbitrarily set in many studies as 50% to 75% repigmentation [13]. The prognosis of the disease is very slow and depends on the patient's skin condition and the triggering factors such as stress level. The patients, as they are not aware of this fact, prematurely discontinue the treatment and switch on to another doctor, again leading to noncompliance [14].

Studies aiming to investigate clinico-epidemiological profile of vitiligo are timely and very critical, particularly for patients living in developing countries where the disease is not well-recognized, which significantly affects their clinical outcome. To the best of our knowledge, little is known about the status of Vitiligo in Ethiopia, in which the current study aimed to generate inferences for the study settings, which are the selected dermatologic clinics in Mekelle city, Ethiopia. Therefore, this study assessed the clinico-epidemiological profile and treatment pattern of Vitiligo in Mekelle city, Ethiopia.

## 2. Methods and Patients

An institutional-based cross-sectional study was conducted at three dermatologic clinics, namely, Ayder Comprehensive Specialized Hospital which is governmental hospital and two other private clinics called Dr. Welderufael Alemayehu dermatologic clinic and Yohannes dermatologic clinic. All these clinics were conveniently selected as study centers because these were the well-known clinics which serve for most of the patients with dermatologic complaints in Mekelle city, Northern Ethiopia.

A two-phased study was conducted on two different sample populations. The first phase was a retrospective study aiming at determining the prevalence of vitiligo. For this purpose, 403 dermatological patients were randomly selected from the total dermatological cases presented to the dermatologic clinics in the year 2017 to 2019. The second phase was aiming at describing the clinico-epidemiological profile and treatment pattern of vitiligo. This phase included cases of vitiligo that were found in the first phase as well as other vitiligo cases that were found from a two months' period prospective study which was intentionally conducted to add more vitiligo cases. Thus, the second phase study included a total of seventy nine patients with vitiligo provided that fifty-three of them were brought from the first phase study and twenty-six were found in the prospective study. Individuals who were seriously ill during the study period were excluded from the study.

A questionnaire was initially developed using a relevant literature search and incorporating all necessary variables including sociodemographic, epidemiological, and clinical data. The questionnaire was developed in English language initially, and then it was translated to the local language which is Tigrigna. The data collection format was pretested in 5% samples, and modifications were done accordingly before the actual data collection was commenced.

For data collection purpose, six nurses who had special training on skin diseases were recruited to smoothly facilitate the process. Medical records were employed to extract clinical data, and an interview was also conducted to collect epidemiological data. The prescriptions of the individual patients were collected to assess the therapeutic management pattern.

Data were entered using Epi Data® version 3.1 and analyzed using SPSS® version 21. Frequency distributions and percentages were utilized to describe categorical variables whereas measure of central tendencies and dispersion were used to describe continuous variables. For categorical variables, comparisons between groups were performed using the chi-squared test. A *p* value at or less than 0.05 was considered statistically significant.

## 3. Results

### 3.1. Prevalence of Vitiligo

Of the 403 randomly sampled dermatological patients, fifty three (53) of them were vitiligo patients giving a prevalence of 13.15%. Further disaggregation by healthcare facility showed that a relatively higher vitiligo cases presented to the dermatologic clinic of Ayder Comprehensive Specialized Hospital (22, 5.45%) followed by Yohannes dermatologic clinic (19, 4.71%) and Dr. Welderufael Alemayehu dermatologic clinic (12, 2.97%).

### 3.2. Epidemiological Profile of Vitiligo

**Figure 1:**
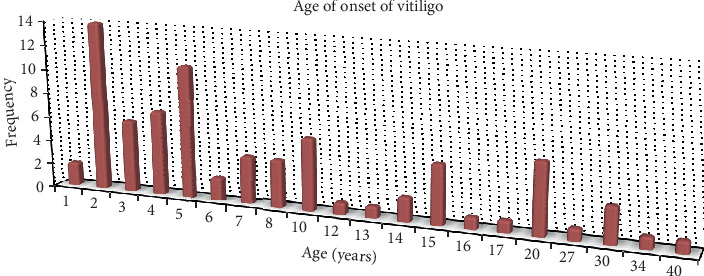
Magnitude of vitiligo by age at onset of the disease in the selected dermatologic clinics in Mekelle city, Ethiopia, from the years 2017 to 2019.

Seventy-nine patients with vitiligo were included in the final analysis regarding clinico-epidemiological profile treatment pattern of vitiligo. Of the seventy-nine patients, about two in three (50, 63.3%) of them were males. It was found in all age groups ranged from three to eighty years, and the dominant (44, 55.7%) cases were adults (20–59 years). The median age at onset of the disease was 5 years, and the peak age of incidence was the first decade of life (Figure 1). Age-standardized distribution of vitiligo was similar in both sexes up to the age of 60 years, whereas few female cases were (2, 18.2%) found above the age of 60 years. This had no statistically significant difference (*p*=0.362) (Table 1). Nearly three-fourths (60, 75.9%) of the cases were urban residents. The employed-to-unemployed ratio of the cases was almost equal (1 : 1.07). Similar numbers of cases were found among all occupational types. About one-third (27, 34.2%) of the cases had been substance abusers giving sixteen (59.3%) alcohol users and eleven (40.7%) smokers. Details about epidemiological profile of vitiligo are found in Table 2.

### 3.3. Clinical Profile of Vitiligo

The average duration of vitiligo was 3.5 years, and cases with chronic form of the disease (>1 year) were preponderant (48, 60.8%). Nearly one-third (25, 31.6%) of the cases had positive family history of vitiligo. Patients with positive family history in a first-degree relative were dominant (22, 88%), whereas none of them had positive family history on third-degree relative. Moreover, five (6.3%) patients had history of vitiligo on their siblings.

The commonest (39.2%) clinical form of vitiligo was vulgaris type followed by focal (26.6%), segmental (20.3%), acrofacial (5.1%), universal (5.1%), and mucosal (3.8%). It was surprising that universal type of vitiligo was found only among male patients (Table 3). However, distribution of vitiligo types was not significantly associated with gender difference (*p*=0.276).

**Figure 2:**
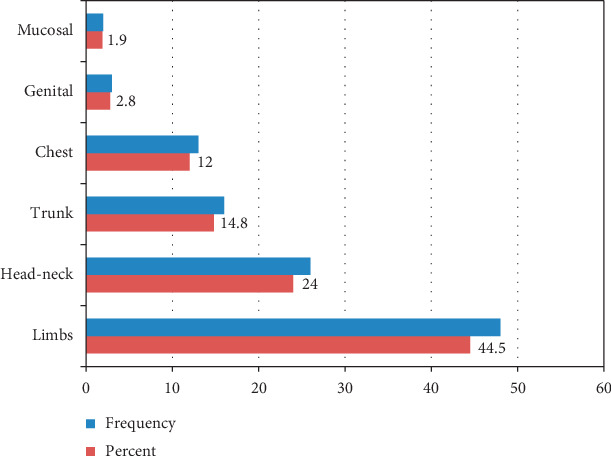
Sites of onset of vitiligo in the selected dermatologic clinics in Mekelle city, from the years 2017 to 2019.

The most commonly affected site were limbs (48, 44.5%) followed by the head and neck (26, 24%), trunk (16, 14.8%), chest (13, 12%), genital (3, 2.8%), and mucosal areas (2, 1.9%) (Figure 2). However, sites of vitiligo were not significantly associated with the age (*p*=0.129) and sex (*p*=0.099) of the cases.

Vitiligo was precipitated by emotional disturbance in majority of the cases (24, 33.8%) followed by physical trauma (23, 32.4%), sunburn (18, 25.4%), and chemicals (6, 8.4%).

Majority (53, 67%) of the patients had associated systemic diseases. The most common was asthma (15, 28.3%) followed by hypertension (12, 22.6%), diabetes mellitus (10, 18.9%), alopecia areata (9, 17%), and thyroid disease (7, 13.2%) (Table 3).

### 3.4. Treatment Trend of Vitiligo

Majority (83.5%) of the cases visited the dermatologic clinic when they noticed the appearance of white patches. However, a few (16.5%) of them visited traditional healers. The average clinic visit was two times, and the peak visit was seven times. More than three clinic visits were majorly recorded by cases affected on their genital areas (66.7%) followed by mucosal (50%) and trunk (50%) sites. The number of clinic visits was also found to increase with age. Based on that, children and adolescents had not more than two visits, whereas 45.5% and 81.8% of the adults and elderlies, respectively, had more than three visits (Table 4).

The average number of medications per prescription was two. Of the topical medications for vitiligo, corticosteroids were found in majority (75.5%) of the prescriptions followed by sun screen lotion (24.5%). On the other hand, oral vitamin E was the majorly prescribed systemic medication (43.3%) followed by multivitamin (27.8%), prednisolone (23.3%), and vitamin B complex (5.6%). Unfortunately, phototherapy and surgical repigmentation were not done in any of the cases.

## 4. Discussion

The prevalence of vitiligo was found to be high. This was higher than reports from India (1.3%) (6250 outpatients in five months' period) [15] and Nigeria (4.96%) (1,652 from May 2005 to April 2009) [16]. On one hand, the small number of population of our study might have overestimated the prevalence of vitiligo. On the other hand, varying ethnic backgrounds of the population residing in different geographic regions with varying environmental conditions may also contribute to the wide variation in the prevalence of vitiligo.

In the present study, majority of the cases were presented to Ayder Comprehensive Specialized Hospital, which is a governmental hospital, than the private dermatologic clinics. This might be explained by the higher cost of treatment in the private clinics.

Males were found to be more affected than females. This was in agreement with various studies [17, 18]. However, the disease generally has no predilection for any sex as was noted by Dave et al. [19]. The observed male preponderance in our cases is presumably for a reason that males being bread earners in contemporary societies, they might be involved in highly risky works which leads to physical trauma as was noted in 69.9% of our male cases.

The commonest age at onset of vitiligo in this study was the first decade of life. This was similar to a report from Sudan [20] and China [21]. Like the study done in Sudan [20], onset of vitiligo at birth was not detected in this study too.

Vitiligo vulgaris was the most common clinical form (31, 39.2%). This agreed with the studies done in Manipal hospital, India [15], South Tunisia [22], and Turkey [1], whereas acrofacial vitiligo was noted to be the most common form in studies performed in India [23] and Libya [24]. However, with the present state of our knowledge, it is difficult to comprehend the mechanisms and determinants underlying varying clinical patterns of vitiligo seen in different patients [15].

Majority of our cases were urban dwellers. This concurred with a study conducted in India [15]. Environmental pollution and highly stressed life among urban dwellers might be a due risk to develop vitiligo.

A positive family history of vitiligo was seen in nearly one-third (31.6%) of the cases. This was in agreement with the study conducted in Sudan [20] and with a study conducted by Gopal et al. [17]. However, it is higher than a finding reported by Ki [25]. A common practice of consanguineous marriage in our society might be a reason for an incidence of the disease among family members.

Emotional disturbance and physical trauma were majorly reported as possible precipitating factors of vitiligo. This was similarly reported by the studies conducted in Moshi, Tanzania [26], and Khartoum, Sudan [20]. Such precipitating factors might be common in developing countries due to the poor quality of living.

The limbs were the most commonly reported sites at the onset of vitiligo. It was concordant with a finding given by Akrem et al. [22]. In contrary, the face was the most common site in a study done by Handa and Kaur [27]. The exact significance of this observation is difficult to appreciate. Nevertheless, the exposed and trauma-prone sites such as the lower limbs and hands may develop vitiligo lesions more easily in genetically predisposed individuals.

The most commonly found associated systemic disease was asthma. This was followed by diabetes mellitus, alopecia areata, and thyroid disease. Another interesting study conducted by Handa and Kaur has revealed similar finding [27]. This might be due to the fact that autoantibodies against different organ systems may also affect the melanocytes.

In this study, some of the cases were alcohol users and smokers. Thus, the chemical content of the substances might dispose persons to be affected by such an immunity-driven disease.

When white patches first appeared, majority of our cases visited healthcare facilities. However, a few of them visited traditional healers. Health-seeking behavior of the patients might be dependent on their perception towards the disease.

Moreover, higher clinic visit was seen among patients having genital or mucosal areas affected by vitiligo. This may be due to the perception that the organs could easily be damaged by vitiligo and may end up with serious complications. Another finding was that adults and elderlies were found to have more frequent clinic visits. As age increases, patients might develop better awareness towards the disease.

In the present study, patients with vitiligo were treated with topical and systemic steroids, sun screen lotion, and vitamins. However, phototherapy and surgical repigmentation therapy were not part of the vitiligo management. Generally, management options of vitiligo were very limited in Ethiopia.

## 5. Conclusion

The prevalence of vitiligo was found to be high. The clinico-epidemiological profile of vitiligo in Ethiopia was similar with that found globally. However, treatment options of vitiligo were very limited in Ethiopia.

## Figures and Tables

**Table 1:** Vitiligo distribution by age and sex of participants in the selected dermatologic clinics in Mekelle city, Ethiopia, from the years 2017 to 2019.

Age (years)	Sex		*p* value
	Male (*n* (%))	Female (*n* (%))	
≤12	4 (44.4)	5 (56.6)	0.362
13–19	9 (60)	6 (40)	
20–59	28 (63.6)	16 (36.4)	
≥60	9 (81.8)	2 (18.2)	

**Table 2:** Epidemiological profile of vitiligo in the selected dermatologic clinics in Mekelle city, Ethiopia, from the years 2017 to 2019.

Variable	Frequency (percent)
Gender	
Male	50 (63.3)
female	29 (36.7)

Age (years)	
<12	9 (11.4)
13–19	15 (19.0)
20–59	44 (55.7)
>60	11 (13.9)

Marital status	
Single	42 (53.2)
Married	30 (38.0)
Divorced	4 (5.1)
Widowed	3 (3.7)

Religion	
Orthodox	51 (64.6)
Muslim	28 (35.4)

Residence	
Urban	60 (75.9)
Rural	19 (24.1)

Type of occupation	
Professionals	11 (13.8)
Craft and related workers	10 (12.5)
Plant and machine operators	8 (10)
Elementary occupations	8 (10)

Level of education	
Higher	31 (39.2)
Secondary	18 (22.8)
Primary	22 (27.8)
No schooling	8 (10.2)

Substance habit	
Yes	27 (34.2)
No	52 (65.8)

Type of substance habit	
Smoking	11 (40.7)
Alcohol use	16 (59.3)

**Table 3:** Clinical profile of vitiligo in the selected dermatologic clinics in Mekelle city, from the years 2017 to 2019.

Variable	Frequency (percent)
Duration of vitiligo	
≤1 years	31 (39.2)
a>1 years	48 (60.8)

Family history of vitiligo	
Yes	25 (31.6)
No	54 (68.4)

Degree of family history	
1^st^ degree	22 (88)
2^nd^ degree	3 (12)

Clinical form of vitiligo	
Vulgaris	31 (39.1)
Focal	21 (26.6)
Segmental	16 (20.3)
Acrofacial	4 (5.1)
Universal	4 (5.1)
Mucosal	3 (3.8)

Precipitating factors	
Emotional disturbance	24 (33.8)
Physical trauma	23 (32.4)
Sunburn	18 (25.4)
Chemicals	6 (8.4)

Presence of systemic diseases	
Yes	53 (67)
No	26 (33)

Systemic diseases	
Diabetes mellitus	10 (18.9)
Hypertension	12 (22.6)
Asthma	15 (28.3)
Alopecia areata	9 (17)
Thyroid disease	7 (13.2)

**Table 4:** Number of clinic visits by age and site of vitiligo in the selected dermatologic clinics in Mekelle city, 2017–2019.

Variable	Number of clinic visits *n* (%)	
	2 visits	≥3 visits
Age (years)		
≤12	6 (66.6)	0 (0)
13–19	13 (86.7)	0 (0)
20–59	44 (100)	20 (45.5)
≥60	11 (100)	9 (81.8)

Site of vitiligo		
Limbs	46 (95.8)	22 (45.8)
Head-neck	24 (92.3)	10 (38.4)
Trunk	15 (93.9)	8 (50)
Chest	12 (92.3)	4 (30.8)
Genital	3 (100)	2 (66.7)
Mucosal	2 (100)	1 (50)

## Data Availability

The data used to support the findings of this study are included within the article.
